# A novel computational strategy for defining the minimal protein molecular surface representation

**DOI:** 10.1371/journal.pone.0266004

**Published:** 2022-04-14

**Authors:** Greta Grassmann, Mattia Miotto, Lorenzo Di Rienzo, Giorgio Gosti, Giancarlo Ruocco, Edoardo Milanetti

**Affiliations:** 1 Università di Bologna, Bologna, Italy; 2 Center for Life Nano & Neuroscience, Italian Institute of Technology, Rome, Italy; 3 Department of Physics, Sapienza University, Rome, Italy; Brooklyn College of the City University of New York, UNITED STATES

## Abstract

Most proteins perform their biological function by interacting with one or more molecular partners. In this respect, characterizing local features of the molecular surface, that can potentially be involved in the interaction with other molecules, represents a step forward in the investigation of the mechanisms of recognition and binding between molecules. Predictive methods often rely on extensive samplings of molecular patches with the aim to identify hot spots on the surface. In this framework, analysis of large proteins and/or many molecular dynamics frames is often unfeasible due to the high computational cost. Thus, finding optimal ways to reduce the number of points to be sampled maintaining the biological information (including the surface shape) carried by the molecular surface is pivotal. In this perspective, we here present a new theoretical and computational algorithm with the aim of defining a set of molecular surfaces composed of points not uniformly distributed in space, in such a way as to maximize the information of the overall shape of the molecule by minimizing the number of total points. We test our procedure’s ability in recognizing hot-spots by describing the local shape properties of portions of molecular surfaces through a recently developed method based on the formalism of 2D Zernike polynomials. The results of this work show the ability of the proposed algorithm to preserve the key information of the molecular surface using a reduced number of points compared to the complete surface, where all points of the surface are used for the description. In fact, the methodology shows a significant gain of the information stored in the sampling procedure compared to uniform random sampling.

## Introduction

Interactions between biomolecules play a fundamental role for most cellular processes, from DNA replication to protein degradation [1–3]. Since it has been estimated that over 80% of proteins operate in molecular complexes [4], many computational approaches have been developed for investigating and/or predicting protein–protein interactions (PPIs) in physiology and pathology [5–10]. In this scenario, the shape complementarity between interacting regions plays a fundamental role because at short distances, it dictates the stabilizing role exerted by van der Waals interactions: thus, the shape of local surface regions has a key role in predicting the ability of a protein to bind its molecular partner [11]. From this point of view, the definition of the molecular surface plays an important role [12–17]. Many algorithms have been designed in order to build the molecular surface at different resolution scales partner [18], typically considering a probe radius up to 5 Å, which is approximately the size of a large solvent molecule. The variation in the size of the probe molecule affects the level of detail of the molecular surface [12] as much as the density of points used in its representation.

Computational methods that aim at the identification of hot-spots and/or binding regions often perform extensive samplings of molecular surface portions, or patches [8, 19, 20]. Intuitively, the more the surface is sampled the more the reaching for hot-spot is accurate. Similarly, the higher the number of different points used to represent the surface the higher the level of detail of the molecular shape.

However, time and computational costs limit both the resolution of the surface and the number of patches that can be sampled, especially for large protein complexes and/or in analyses that involve a big set of surfaces, like for example molecular dynamics data. Although several methods have been developed over the past decades to optimize the computational calculation of any molecular surface, the search for optimal techniques still remains a challenge today [21–23].

Here, we present a new method for reducing the molecular surface, maximizing the overall information of the protein shape, and minimizing, in principle, the number of total surface points. The basic idea of the proposed new algorithm is the selection of molecular surface points according to the local roughness (that is the degree of complexity of shape of each surface region): increasing the sampling in high roughness regions and decreasing the sampling in the more flat ones. In particular, we define a sampling probability that depends on the local roughness of the surface. We discuss the performance of the algorithm as a function of several parameters, evaluating the ability of the algorithm to describe locally portions of the surface in comparison to uniform random sampling. In order to evaluate the ability of the reduced molecular surface to capture the information of the complete surface, we define a descriptor based on the local characterization of the molecular surface patch shape. Indeed, to evaluate the resulting representation of the surface, we compare the shape similarity between a portion of the surface obtained from the complete surface of the protein and the same portion of the surface obtained via our algorithm.

In order to study the gain of the proposed algorithm in terms of information preserved in the reduced version of the molecular surface, we compare the description of the complete surface also with random sampling, which represents the approach of trivial reduction of each molecular surface by decreasing, without criteria, the density of the number of points in space.

While part of the information concerning the interaction is encoded in various chemical features, it has already been discussed how the shape of local surface regions has a key role in predicting a protein ability to bind its molecular partner [11, 24]. This is at the core of the development of the 2D Zernike polynomial expansion [8] as a method to find the protein-protein binding regions. This method could in principle be adapted to other properties (such as electrostatics or hydrophobicity) besides curvature since they can be described with numerical values that can be assigned to each surface point [25, 26], and the Zernike expansion can be applied to any function. Nonetheless, in this work the algorithm is used to maximize the information concerning only the overall shape of the molecule. More specifically, the method is based on the description of each portion of the molecular surface by expanding the well-exposed molecular surface patches in terms of 2D *Zernike* polynomials, in order to be able to measure the geometrical similarity or complementarity between interacting proteins, with a lower computational cost than its 3D version [27–32].

We show that our proposed sampling reduces the number of considered points, minimizing the loss of information about the protein surface shape. To propose a generically valid set of parameters, we estimate the optimal sets for 70 proteins and compute their average values, which are: *β* = 0, *γ* = 4, *δ* = 5. By comparing the percentage of points sampled with each surface’s best set and with these average values, we show that these last ones mostly results in a number of points close to those selected with the specific optimal parameters.

Finally, to test the general validity of our method in a biologically relevant case (i.e. the protein-protein interaction), we study a subset of protein complexes among the ones used in [8] and evaluate the performance of the Zernike-based method in recognising the interacting regions of protein structures. In particular, we analyze the ability of the proposed method to characterize binding sites of these protein complexes, the structure of which has been experimentally resolved. For this purpose, we select the interacting regions of four protein surfaces and sample them with our proposed algorithm.

By analyzing how the total and sampled points distribute in the two-dimensional principal components (PCs) of the space spanned by the vectors of the Zernike invariants associated to each surface patch, we show that the subsets of points sampled using the best parameters’ set fully cover the same portion of the essential space occupied by the whole bunch of surface points.

## Materials and methods

### Protein structure and computation of the molecular surfaces

The protein used in the first part of this work is TDP-43, residues 209-269 (PDB id: 4bs2). The protein was equilibrated using GROMACS 2019.3 [33], and is going to be called A1 in the following discussion. For the optimization and testing of the parameters’ selection we used 70 proteins complexes experimentally solved in complex in X-ray crystallography, taken from [8].

The solvent-accessible surfaces are calculated with DMS [34], with a density of 5 points per *Å*^2^ and a water probe radius of 1.4 *Å*. For each surface point, we calculated the unit normal vector with the flag *−n*.

### Zernike 2D procedure

The molecular surface we obtain with DMS is represented by a set of points in the three-dimensional space. The first step of the 2D *Zernike* algorithm is to select from the surface a patch Σ, defined as the set of surface points included in a spherical region having radius *R*_*zernike*_ = 6 Å and centered on one point of the surface. The points not directly connected to that region of the surface (for example coming from a protuberance included in the sphere) are removed. Once the patch has been selected, the mean vector of the normal vectors of the patch points is computed and oriented along the *z*-axis. Thus, given a point *C* on the *z*-axis, the angle *θ* is defined as the largest angle between the *z*-axis and a secant connecting *C* to any point of the surface Σ. *C* is then set so that *θ* = 45° and each surface point is labeled with its distance *r* from *C*. As a next step, a square grid that associates each pixel with the mean *r* value calculated on the points inside it is built. The gap of pixels where no point of the surface has been projected are filled by using the average value of the surrounding pixels.

Such a 2D function can now be expanded on the basis of the *Zernike* polynomials.

Indeed, each function of two variables *f*(*r*, *ψ*) defined in polar coordinates inside the region of the unitary circle (*r* < 1) can be decomposed in the *Zernike* basis as

$$\begin{array}{c} f\left(r,\psi \right)=\sum_{n^{\prime }=0}^{\infty }\sum_{m=0}^{n^{\prime }}c_{n^{\prime }m}Z_{n^{\prime }m}\left(r,\psi \right), \end{array}$$ (1)

with

$$\begin{array}{c} c_{n^{\prime }m}=\frac{n^{\prime }+1}{\pi }\int_{0}^{1}dr\;r\int_{0}^{2\pi }d\psi Z_{n^{\prime }m}^{*}\left(r,\psi \right)f\left(r,\psi \right) \end{array}$$ (2)

and

$$\begin{array}{c} Z_{n^{\prime }m}=R_{n^{\prime }m}\left(r\right)e^{im\psi }. \end{array}$$ (3)

*c*_*n*′*m*_ are the expansion coefficients, while the complex functions *Z*_*n*′*m*_(*r*, *ψ*) are the *Zernike* polynomials. The radial part *R*_*n*′*m*_ is given by

$$\begin{array}{c} R_{n^{\prime }m}\left(r\right)=\sum_{k=0}^{\frac{n^{\prime }-m}{2}}\frac{{\left(-1\right)}^{k}\left(n^{\prime }-k\right)!}{k!(\frac{n^{\prime }+m}{2}-k)!(\frac{n^{\prime }-m}{2}-k)!}. \end{array}$$ (4)

Since for each couple of polynomials, it is true that

$$\begin{array}{c} \langle Z_{n^{\prime }m}\left|Z_{n^{\prime \prime }m^{\prime }}\right\rangle =\frac{\pi }{n^{\prime }+1}\delta_{n^{\prime }n^{\prime \prime }}\delta_{mm^{\prime }}, \end{array}$$ (5)

the complete set of polynomials forms a basis, and knowing the set of complex coefficients *c*_*n*′*m*_ allows for a univocal reconstruction of the original patch.

The norm of each coefficient *z*_*n*′*m*_ = |*c*_*n*′*m*_| constitutes one of the *Zernike* invariant descriptors.

### Similarity evaluation

Once a patch is represented in terms of its Zernike descriptors, the shape relation between that patch and another one can be simply measured as the Euclidean distance between the invariant vectors. The relative orientation of the patches before the projection in the unitary circle must be considered. In fact, if we search for similar regions we must compare patches that have the same orientation once projected in the 2D plane, i.e. the solvent-exposed part of the surface must be oriented in the same verse for both patches, for example along the positive z-axis. On the other hand, if we are looking for complementarity (as for searching the binding regions in a complex) the patches have to be oriented in opposite verses.

### Identification of the binding regions and principal component analysis

For each of the 70 protein complexes sampled from [8] and reported in Table 2, the binding regions between the couples of proteins were defined as the sets of surface points whose distance with respect to the partner was lower than 3 *Å*. Finally, for each set, we compute its geometrical center and then consider as binding region the patch with radius 6 *Å* centered on it.

From each point of the identified binding regions, we evaluated the set of Zernike invariants and performed a principal component analysis (PCA) in which the starting matrix consisted of 121 columns (the number of invariants) and a number of rows equal to the number of points of the patch.

## Results

The proposed method consists of a sampling suitably designed for selecting with greater probability surface points belonging to regions of high roughness. Indeed, when choosing where to select the patches, a compromise between a too sparse sampling (which would loose some regions of the surface) and a too dense one (which would add no information and would instead increase the computational time) must be found.

To this end, we define a function used for sampling the original surface: we use a method based on the 2D formalism of the *Zernike* polynomials to evaluate the ability of the algorithm to correctly approximate local regions of molecular surfaces with respect to a uniform random sampling. In the following Sections, we describe in detail the designed algorithm, the method we used as test based on the 2D *Zernike* formalism, and the results obtained by varying the parameters.

For an in-depth study of the proposed algorithm we consider different cases of application to a single surface (namely a fragment of TDP-43 that we are going to call A1). As a next step, to make this method applicable to a variety of surfaces, we apply it on a set of 70 protein surfaces. For each surface we test different parameters’ combinations, selecting the best ones. This allows us to propose a set of mean parameters to be used for any given protein.

### New roughness-dependent sampling

To begin with, we numerically represent the molecular surface with a set of N points in the 3D space (the discretization of the surface). For each point *i*, we evaluate the exiting normal vector, ${\bar{v}}_{i}$, to the surface, originating from *i*. Next, we evaluate the local roughness of the molecular surface by looking at the relative orientation of the normal vectors with respect to each point *i*.

**Fig 1:**
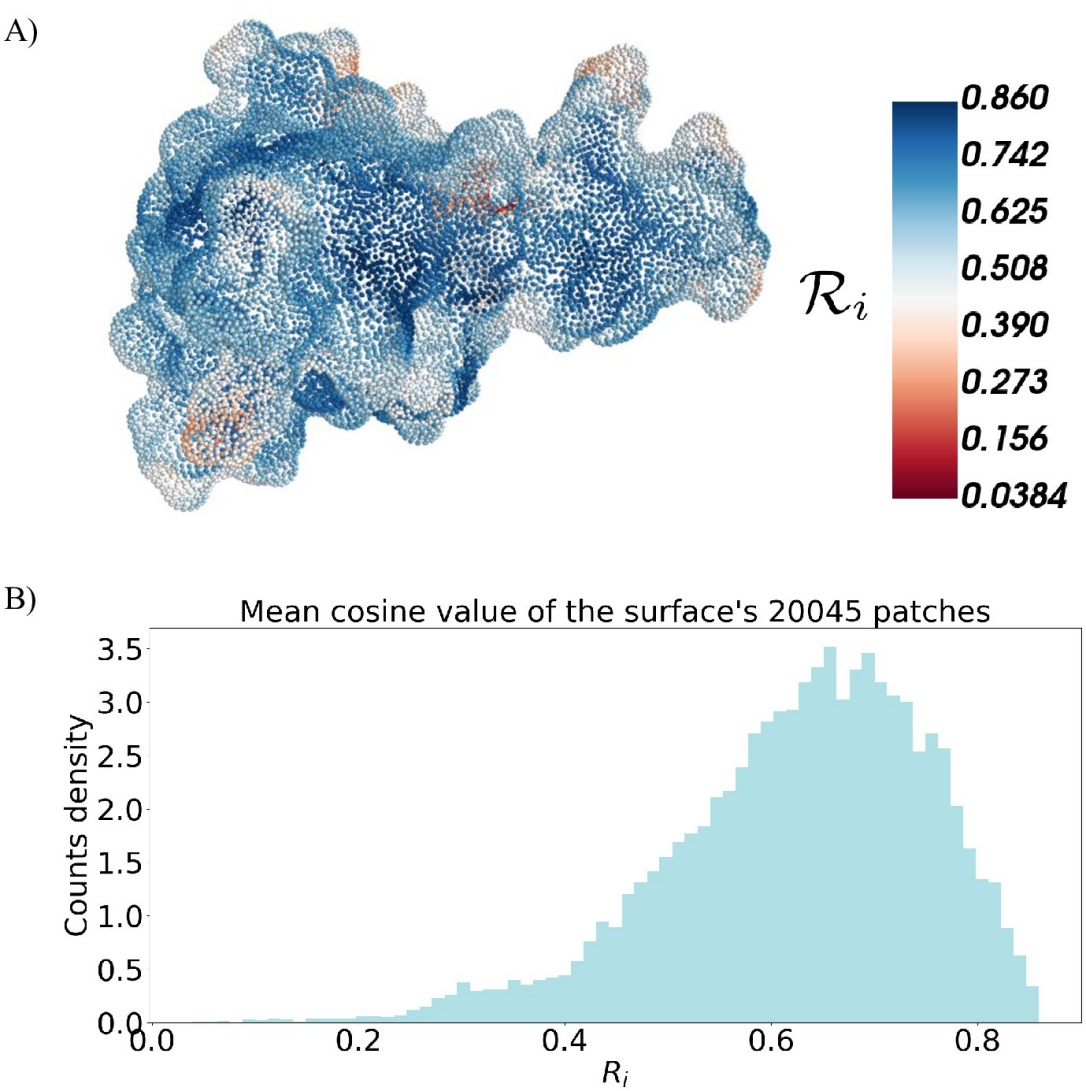
Local roughness of the patches. A) Discretized representation of a molecular surface of the TDP-43 fragment 209-269 (PDB id: 4BS2). Each point of the surface is coloured according to the local roughness value, $R_{i}$. B) Distribution of the roughness $R_{i}$ found for each point *i* of the considered surface.

To do so, starting from each point *i*, we define a patch including all the surface points within a sphere of radius *R*_*patch*_ centered on the point *i*. We calculate the roughness of each patch as the mean of the cosines of the angles formed by the normal vectors associated to each of the *n*_*p*_ points of the patch and the average normal vector:

$$\begin{array}{c} R_{i}=\frac{1}{n_{p}}\sum_{j=1}^{n_{p}}\text{cos}\left(\theta_{ij}\right) \end{array}$$ (6)

with $\text{cos}\left(\theta_{ij}\right)=\frac{{\bar{v}}_{i}\cdot {\bar{v}}_{j}}{|{\bar{v}}_{i}\left|\right|{\bar{v}}_{j}|}$ and ${\bar{v}}_{i}=\frac{1}{n_{p}}\sum_{j}{\bar{v}}_{j}$. Fig 1A shows the molecular surface for a case-of-study protein, TDP-43 (PDB id: 4BS2) residues 209-296, colored according to the local roughness. Being a mean of cosines, the roughness ranges from zero to one (see Fig 1B). When the considered patch is plane, the mean value of the cosine between the normal vectors of each point *i* of the surface and the mean normal vector of that patch, $R_{i}$, is close to one, while lower values of $R_{i}$ indicate rougher patches.

Then, we associate to each point *j* in the patch centered on a point *i*, the probability to be accepted for the sampling, defined as:

$$\begin{array}{c} p\left(j\right)=\alpha {\left(1-R_{i}\right)}^{\beta }{\left(\frac{r_{i,j}}{R_{patch}}\right)}^{\gamma (1+R_{i})+\delta }, \end{array}$$ (7)

where *r*_*i*,*j*_ is the distance of the point *j* from the center *i*, and *α*, *β*, *γ* and *δ* are parameters that can be optimized to yield different sampling scenarios.

For instance, when *β* = 0, *γ* = 0, and *δ* = 0, we obtain a uniform random extraction, whose total number of points depends on *α*: this parameter determines the fraction (over all the surface’s points) of considered points.

In general, when a patch *i* has a high roughness, more points are needed to describe it. On the other hand when it is more plane we need fewer points, and indeed $\left(1-R_{i}\right)$ becomes smaller. Finally, the center of a patch is always selected, but then to capture the surface’s irregularities we can use as centers for the *Zernike* patches the points further away from it, i.e. the ones with a high value of *r*_*i*,*j*_. By elevating this term to the $\left(1+R_{i}\right)$ we are changing the distribution of sampled points in each patch as a function of that patch roughness.

### Selection of the sampling parameters

Here, we describe the method used to determine how effectively, starting from a sampling (determined by the combination of the parameters *α*, *β*, *γ*, and *δ*), we can describe the original surface.

1. For each combination of these four parameters, we sample from the original total surface a number *n*_*S*_ of points and we define a new surface determined by these *n*_*S*_ points. Then, we extract from the original total surface again *n*_*S*_ points, but this time with a uniform distribution (or random extraction).
2. We select from the total surface *n*_*test*_ points, and define around each of them a region with radius *R* = 6 Å.
3. Next, we associate to each one of these points, *j*, three vectors: *z*_*tot*_(*j*), *z*_*S*_(*j*) and *z*_*R*_(*j*). *z*_*tot*_(*j*) contains the *Zernike* descriptors that describe that patch as defined by all the total points included in it, *z*_*S*_(*j*) describes the patch as defined by the sampled points included in it and *z*_*R*_(*j*) describes the patch as defined by the included randomly extracted points.
4. For each of the *n*_*test*_ patches we compute the distances *Z*_*t*−*S*_(*j*) = *z*_*tot*_(*j*) − *z*_*S*_(*j*) and *Z*_*t*−*R*_(*j*) = *z*_*tot*_(*j*) − *z*_*R*_(*J*). We average all the obtained *Z*_*t*−*S*_(*j*) and *Z*_*t*−*R*_(*j*), and obtain respectively the values *Z*_*t*−*S*_ and *Z*_*t*−*R*_. Since we are considering the description given by all the original points as our “ideal”, for a good sampling we expect the value of *Z*_*t*−*S*_ to be small, and in particular smaller than *Z*_*t*−*R*_.
5. Finally, we compute the difference *d* = *Z*_*t*−*R*_ − *Z*_*t*−*S*_. The best sampling for a surface should result in the maximization of *d*.

Our main objective is to describe the original surface as precisely as possible with a minimum number of points.

### Selection starting from a pre-determined *n*_*S*_

**Fig 2:**
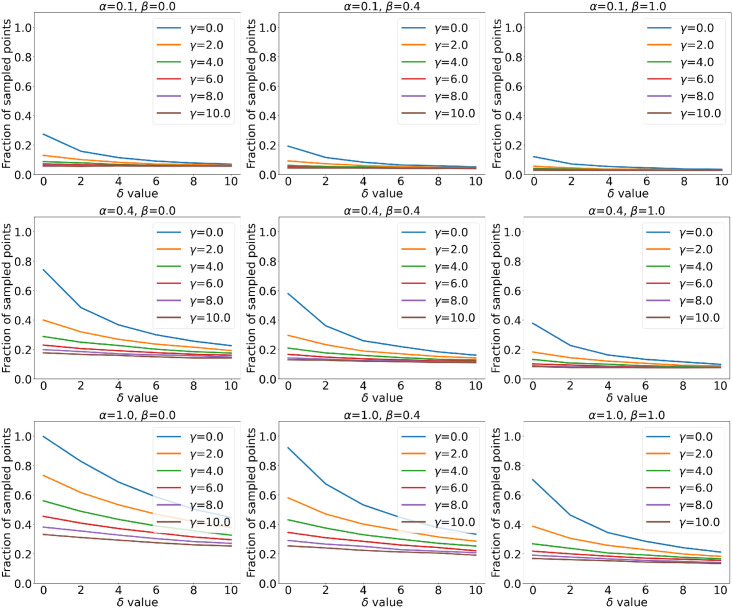
Sampled points fractions for varying parameters. Percentage of surface points that are sampled by varying *α*, *β*, *γ* and *δ*. These plots were obtained after the sampling of the surface of A1.

By varying the four parameters we can observe a wide range of *n*_*S*_. Fig 2 shows an example of how this number changes for changing combinations of parameters. Table 1 shows, for each arbitrarily determined range of *n*_*S*_, the combination of parameters that results in the sampling with the highest *d*.

**Table 1:** Best sampling for each range of selected points (*n*_*S*_).

Range of the sampled points	*α*	*β*	*γ*	*δ*	*d*	*Z*_*t*−*S*_
2.63%–12.34%	0.4	0.2	10.0	10.0	1.61	7.36
12.34%–22.05%	1.0	0.4	10.0	6.0	1.98	6.39
22.05%–31.76%	1.0	0.2	6.0	10.0	2.31	5.85
31.76%–41.47%	1.0	0.0	6.0	4.0	2.46	4.97
41.47%–51.18%	1.0	0.0	6.0	0.0	2.69	4.10
51.18%–60.89%	1.0	0.0	2.0	4.0	2.24	3.24
60.89%–70.60%	1.0	0.2	0.0	4.0	1.70	2.53
70.60%–80.30%	1.0	0.0	2.0	0.0	0.18	1.38
80.30%–90.01%	1.0	0.0	0.0	2.0	-0.14	0.81
90.01%–99.73%	1.0	0.2	0.0	0.0	-0.01	0.27

### Selection in the limit cases

Similarly, we can fix a priori the values of some of the parameters to study some particular forms of the sampling distribution *p*(*j*).

In the following we analyze three peculiar cases:

1. No dependency on the roughness: when *β* = 0 and *γ* = 0, *p*(*j*) does not depend on $R_{i}$, but only on the distance between the center of the considered patch and the considered point.
2. No dependency on the distance from the patch center: when *γ* = 0 and *δ* = 0, *p*(*j*) depends only on $R_{i}$.
3. Steepest sampling function: when *β* = 1 and *γ* = 0 *p*(*j*) has the fastest variation as a function of *δ*. In this situation the best sampling (given these fixed parameters) should be clearly distinguishable.

**Fig 3:**
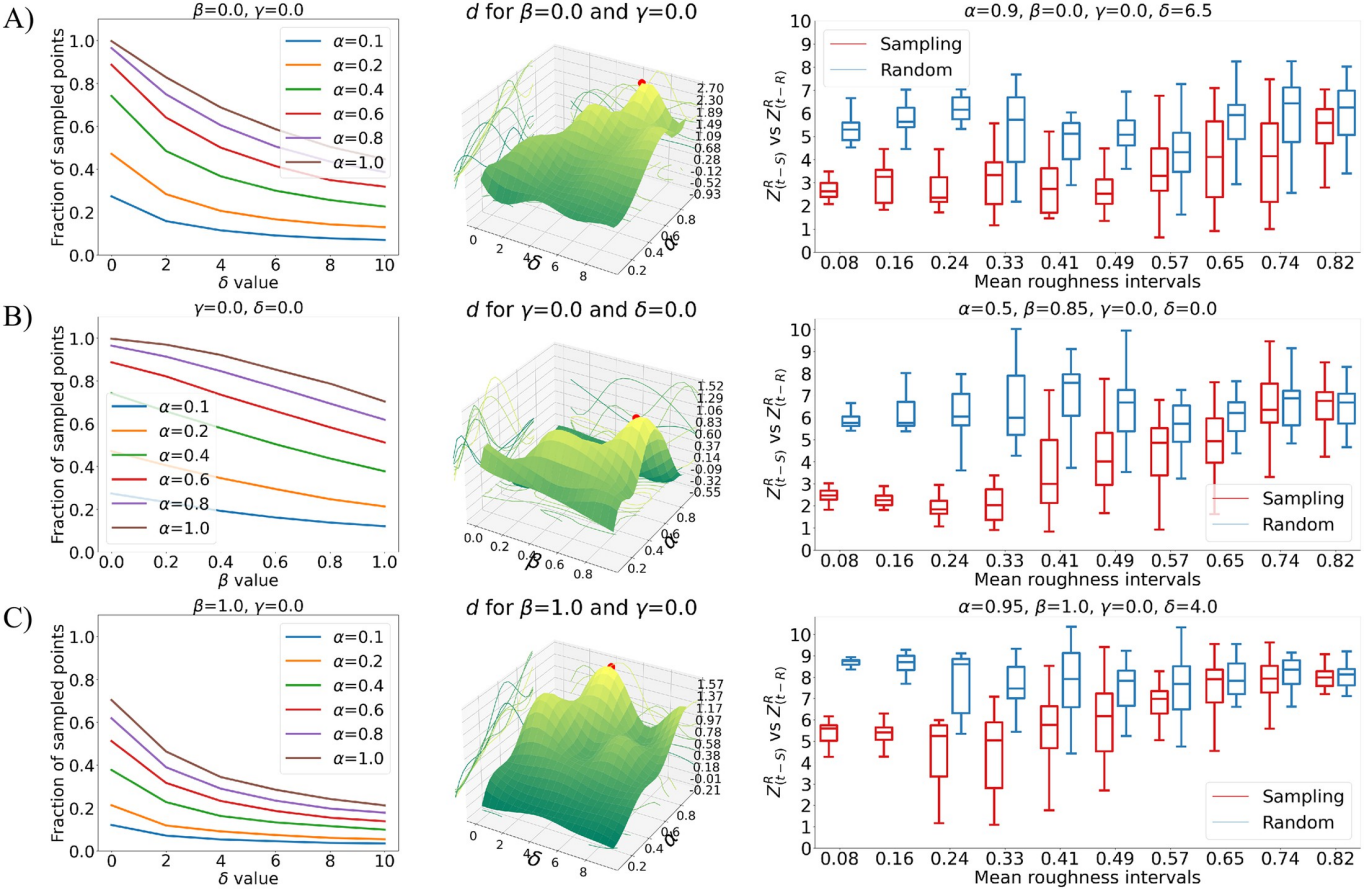
Analysis of the limit cases of the sampling function. A) Results obtained when there is no dependency on $R_{i}$. B) Results obtained when there is no dependency on the distance from the patch center. C) Results obtained with the steepest sampling function. The first column shows the percentage of surface points that are sampled for varying parameters. The second one depicts *d* for varying parameters; these plots are obtained with an interpolation of the effectively computed values, that -when not set to zero or one- are all the combinations of *α* = [0.1, 0.2, 0.4, 0.6, 0.8, 1], *β* = [0, 0.2, 0.4, 0.6, 0.8, 1], *γ* = [0, 2, 4, 6, 8, 10] and *δ* = [0, 2, 4, 6, 8, 10]. The last column shows, for the best parameters combination in each limit case, the box plot for different mean $R_{i}$ of $Z_{t-S}^{R}$ (in red) and $Z_{t-R}^{R}$ (in blue). These plots refer again to the application of this sampling method to the surface of A1.

Fig 3 shows the analyses accomplished in each of the three limit cases. The first useful information is how the number of selected points changes according to the parameters value, because this is a first indication of the performance of that sampling function. Then we can optimize the non-fixed parameters, by looking for the maximization of *d*. Once the best parameters combination has been detected for each case, the differences between the three situations can be better appreciated by looking at what happens for different roughness of the patches. Fig 3 shows for each case the box plot of $Z_{t-S}^{R}$ and $Z_{t-R}^{R}$, divided according to the roughness of the patches they describe. $Z_{t-S}^{R}$ and $Z_{t-R}^{R}$ are defined by averaging *Z*_*t*−*S*_(*j*) and *Z*_*t*−*R*_(*j*) only on the points whose $R_{j}$ falls in a specific interval. The considered patches are extracted (according to their roughness) from the points obtained with the best sampling, in each limit case, of the considered surface.

### Selection without restriction

When there is no restriction on the number of sampled points or on the parameters’ values, we can fix *α* = 1, since it is a multiplicative parameter that causes no variation of the distribution of sampled points between patches with different roughness values. When *α* < 1 we are removing from each patch the same percentage of points, so if we have no requirements about the final *n*_*S*_, this uniform removal of points can be avoided.

Consequently, we are interested in finding the combination of *β*, *γ*, and *δ* that results in the highest *d*. While it is true that a good sampling should result in a high *d* combined with a low *n*_*S*_, the weights that these two components should have in an optimization function will change according to the application and cannot be generalized.

**Fig 4:**
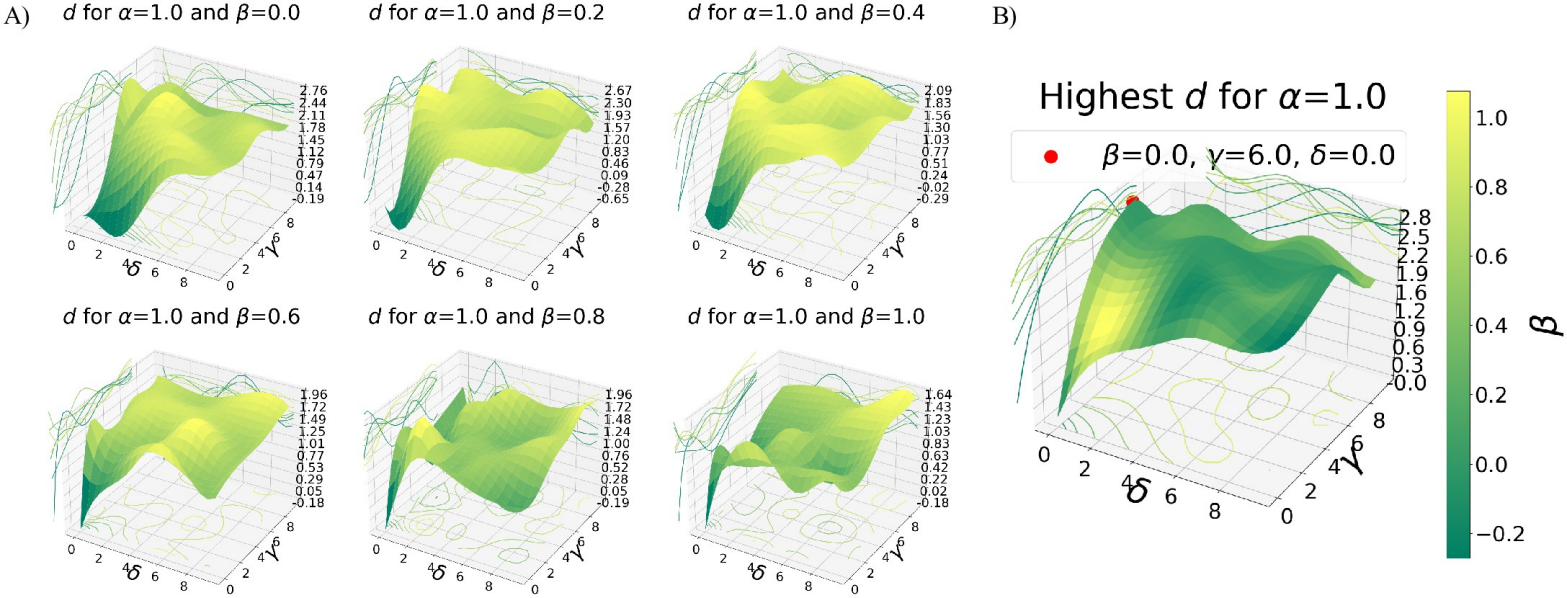
Selection of the absolute best sampling parameters. A) *d* as a function of varying values of *γ* and *δ*. Each plot is obtained with a fixed value for *β*, and for all the plots the same fixed value of *α* = 1.0 is used. The colouring is given by the *d* value. The plotted surfaces are obtained with an interpolation of the effectively computed values, that were obtained with all the possible combination, for each *β* value, of *γ* = [0, 2, 4, 6, 8, 10] and *δ* = [0, 2, 4, 6, 8, 10]. B) For each plot on the left, the point corresponding to the highest *d* is selected. The colouring is given by the *β* value (as described by the color-bar). The plotted surface is obtained from the interpolation of these points, and shows, for all the *δ*-*γ* combinations the value of *β* that will result in the best sampling. The maximum of this surface (red points) corresponds to the best sampling parameters.

**Fig 5:**
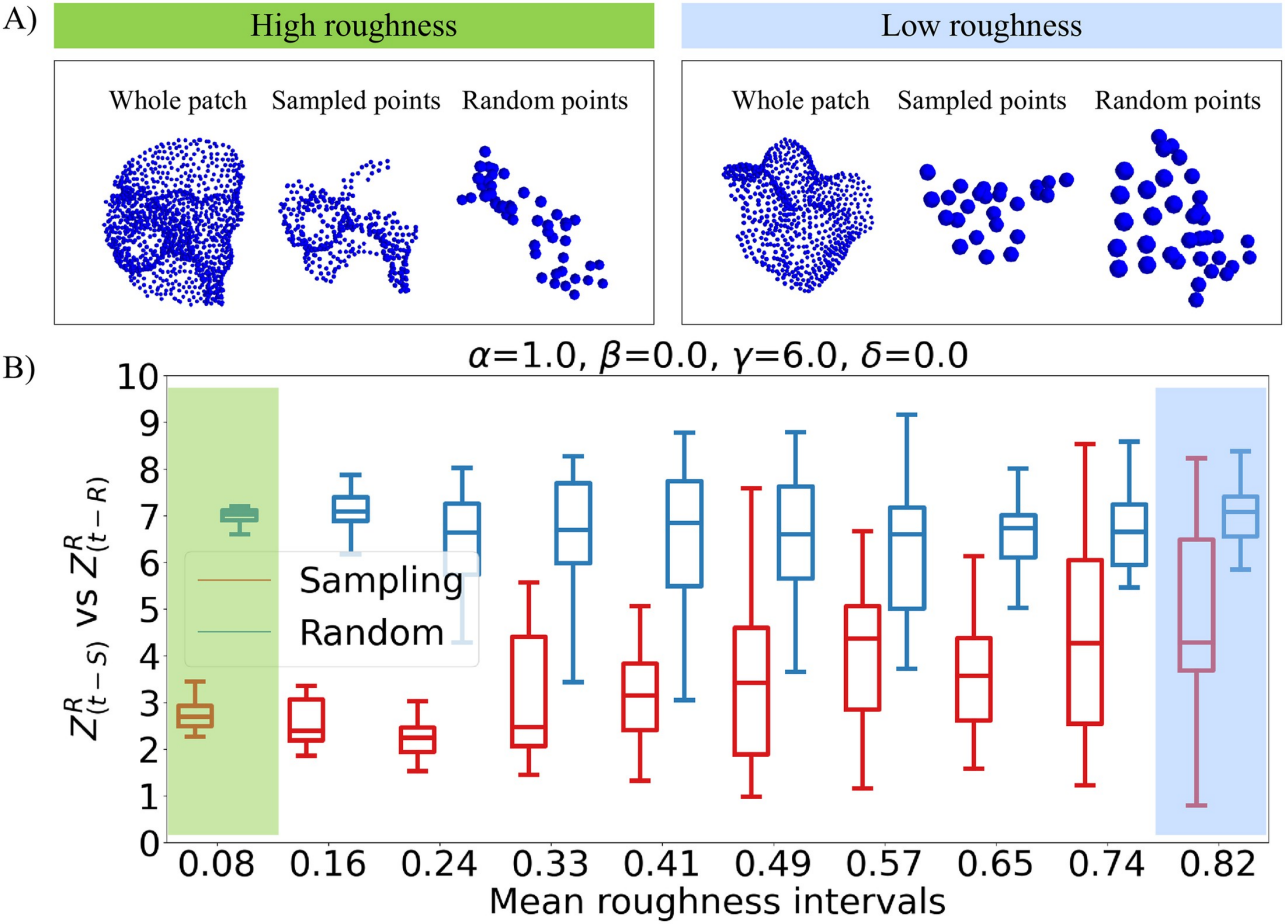
Comparison between the best sampling and the random selection of points for patches with different roughness. A) On the left, an example of how a rough patch is represented with all the points in the surface (*whole patch*), with only the points resulting from the sampling (*sampled points*) and with randomly extracted points (*random points*). On the right, the same three representation cases for a plane patch. B) In red, the box plot for different ranges of patches’ roughness of $Z_{t-S}^{R}$. In blue, the box plot for different ranges of patches’ roughness of $Z_{t-R}^{R}$. This is in the case of a sampling with parameters *α* = 1, *β* = 0, *γ* = 6 and *δ* = 0, whose combination results in the highest value of *d* for the A1 surface.

Fig 4 shows how the best parameters combination is selected: for each *β* we individuate the best values for *γ* and *δ*, then to determine *β* we select among these pairings the one corresponding to the absolute highest *d*. Once the best parameters combination has been detected, we can better understand what exactly makes the sampling better than a random extraction from the total surface of the same number of points. Fig 5 shows for example the box plot of $Z_{t-S}^{R}$ and $Z_{t-R}^{R}$ divided according to the roughness of the patches they describe. As expected, we can see that plane patches (whose $R_{i}$ tends to one) are reconstructed nearly equally with the sampling and the random extraction, whereas for increasing roughness ($R_{i}$ approaching zero) the sampling results in *Zernike* vectors much closer to the ones obtained from the total surface, compared to the random case. That said, it must be remembered that with the sampling we are concentrating the *n*_*S*_ points on the roughest zones. This means that with the sampling the plane patches are described with fewer points than in the random case: the improvement for the rough patches comes from a better surface reconstruction, while for plane patches it derives from the fact that a smaller number of points is used to reach the same reconstruction.

**Fig 6:**
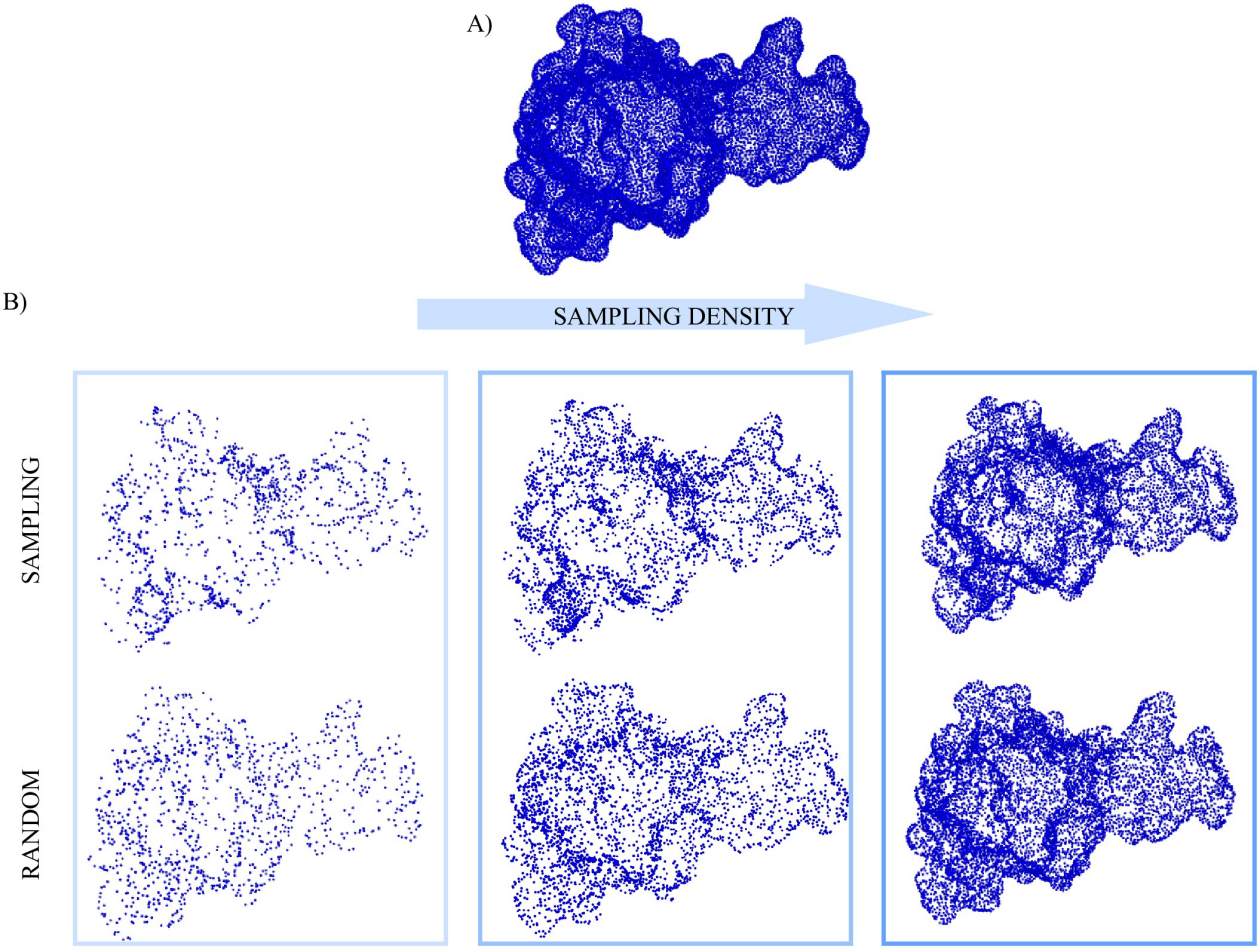
Visualization of the 3D surfaces reconstruction. A) 3D reconstruction of the A1 surface from all its surface points. B) The three columns depict the reconstruction of the same surface, with an increasing sampling density. In each column, the first row shows the reconstruction with a subset of the original points selected with the sampling, whereas the second row shows the reconstruction with a subset that counts the same number of points selected with the sampling, but in this case randomly extracted.

Finally, Fig 6 shows an example of how the surface is described when all its points are considered versus when only some subsets -including increasing number of points- are selected, with the sampling or randomly. When a small subset of points is used to reconstruct the surface, the difference between the sampling or a random extraction of the same number of points is clearly distinguishable. The more points are considered, the more the two selections become similar.

### Optimization of the sampling parameters selection

To make this sampling method of general utility, we identify the best combination of parameters for different cases. Our aim is to provide the user with a list of possible sets of parameters averaged over a large set of different proteins. In particular, we selected 70 protein-protein complexes from [8] and for each of them only a single protein structure is considered for this analysis.

Here, we report for each parameter the mean and standard error over the mean: *β* = 0.026±0.019, *γ* = 0.026±0.019 and *δ* = 0.026±0.019. Interestingly, the *β* parameter appears to be compatible with zero. Finally, we compare the sampling obtained with the optimal parameters and the one with the average parameters. The results are shown in Table 2.

**Table 2:** Comparison between the best and the average set of parameters for 70 proteins.

PDB code	Chain	*β*	*γ*	*δ*	Sampled points	Missed points	PDB code	Chain	*β*	*γ*	*δ*	Sampled points	Missed points
1E6E	A	0	8	2	39	6	2W2X	A	0	4	6	42	2%
1GVM	E	0	6	2	44%	0%	2WY3	A	0.2	0	8	46%	-3%
1GXD	A	0	10	0	39%	7%	2XT2	A	0	0	10	47%	-3%
1IXS	A	0	6	0	47%	-4%	2XTS	A	0	2	8	44%	0%
1K2E	A	0	2	8	46%	-1%	2Y79	A	0	10	4	32%	12%
1K4R	B	0	2	10	42%	3%	2ZBC	F	0	0	8	52%	-9%
1KM1	A	0.2	4	0	51%	-8%	3AXY	B	0	2	10	40%	3%
1LJ2	A	0	4	2	50%	-8%	3DAL	A	0	10	2	35%	9%
1M27	AB	0	10	10	28%	18%	3DFE	A	0	6	6	38%	8%
1MAH	A	0	0	10	46%	-3%	3DRX	B	0	6	0	47%	-4%
1MTO	C	0.2	4	4	40%	3%	3EPN	A	0	2	6	48%	-5%
1NVM	A	0	6	4	40%	4%	3EUA	G	0	0	10	47%	-3%
1OPH	A	0	4	10	38%	8%	3FBI	B	0	6	0	47%	-4%
1PZM	A	0.2	2	4	46%	-6%	3FN2	A	0	2	10	43%	3%
1QGE	D	0	6	2	45%	1%	3FYB	A	0	8	0	40%	3%
1R4C	A	0	10	2	37%	10%	3GJN	B	0	0	10	50%	-3%
1WDX	B	0	6	4	40%	4%	3GOR	C	0	8	0	42%	2%
1XOU	A	0	4	4	46%	-3%	3H35	B	0	2	8	45%	-1%
1YVI	A	0	6	2	43%	0%	3H3B	A	0	2	8	42%	0%
2AEP	A	0	6	4	40%	4%	3HKL	A	0	2	8	44%	0%
2DJF	A	0.2	4	6	40%	7%	3I50	E	0	4	6	43%	2%
2E2P	A	0	10	0	37%	7%	3IJM	A	0	8	2	36%	6%
2GJ3	A	0	4	8	40%	5%	3IQ2	A	0.2	4	2	46%	-2%
2ISJ	C	0	2	10	41%	3%	3IUW	A	0.2	4	2	48%	-1%
2IU9	A	0	6	0	48%	-4%	3IVP	A	0	2	4	53%	-12%
2J98	A	0	4	6	41%	2%	3L0R	A	0	0	8	52%	-9%
2O42	A	0	8	0	41%	3%	3M65	A	0	2	6	49%	-6%
2OZN	A	0	6	0	45%	-4%	3MKR	A	0	6	4	41%	4%
2P10	B	0	2	8	47%	-1%	3MQ0	A	0	4	6	42%	1%
2P49	A	0	2	6	50%	-4%	3MTS	B	0	10	2	32%	10%
2PG1	F	0	2	10	42%	4%	3N0V	A	0	6	0	47%	-4%
2RU4	A	0.2	8	4	32%	12%	3OL3	A	0	8	0	40%	3%
2V9P	G	0	6	6	36%	7%	3R1F	A	0	10	4	33%	12%
2VMK	A	0	6	0	48%	-5%	3RHU	A	0	10	2	33%	10%
2VVX	A	0	4	6	43%	2%	3TUZ	E	0	2	8	47%	-1%

For each protein, the “*β*”, “*γ*” and “*δ*” columns report the best combination of parameters. The “*Sampled points*” column shows the percentage of selected points on the surface, while the last column “*Missed points*” displays the percentage of points that are lost (if the value is positive) or added (if it is negative) when the sampling is performed with the mean parameters *β* = 0, *γ* = 4, *δ* = 5.

Looking at the difference between the percentage of sampled points in the optimal and averaged case (fifth column in Table 2) it seems that the latter is in many case a good approximation of the former. We note that since the sampling best parameters depend on the geometry of the surface, the optimal solution could be to associate the optimal set of parameters to each class of proteins, even if we expect it to be a not trivial relationship worth of future investigations.

### Focus on protein-protein binding regions

**Fig 7:**
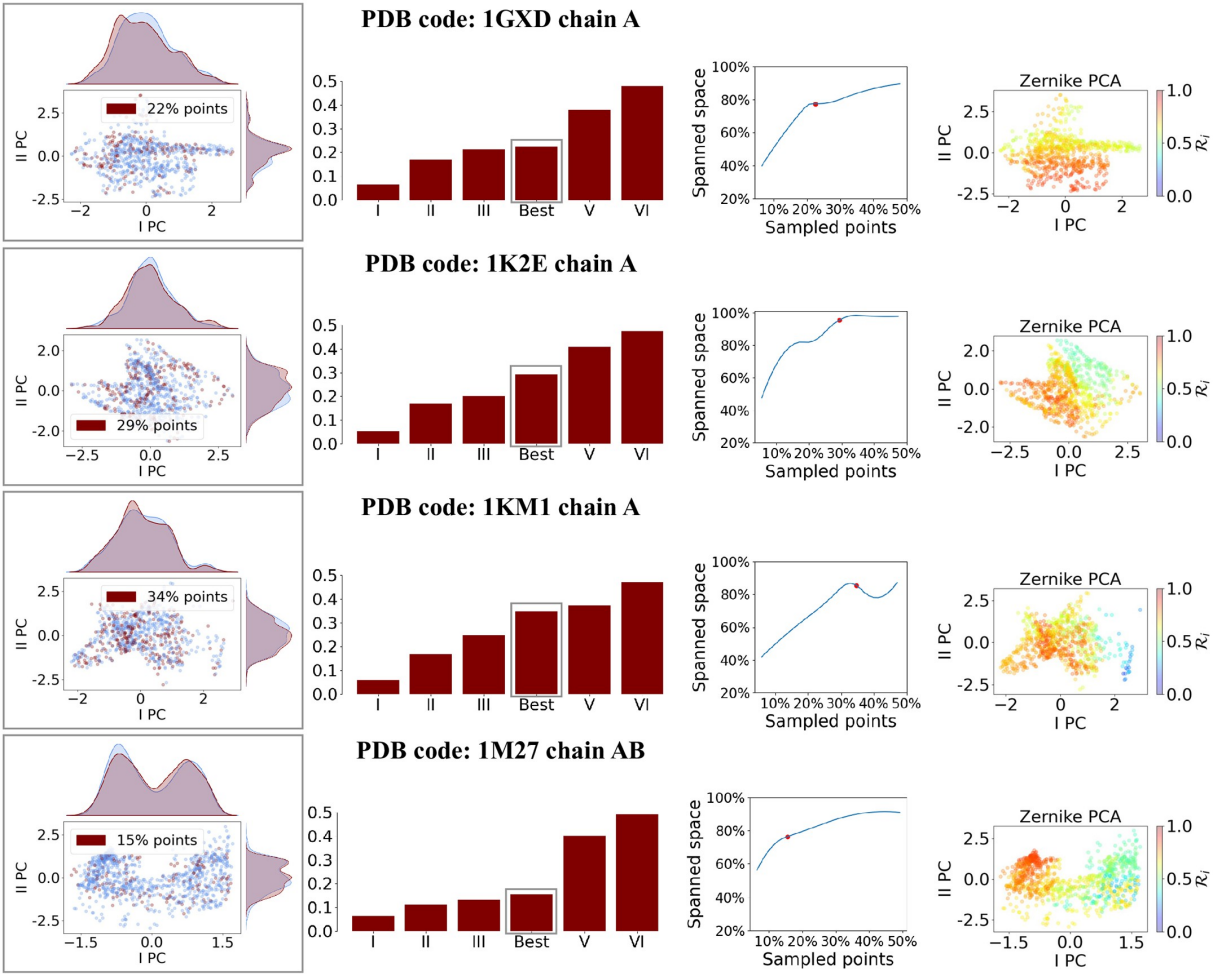
Principal component analysis of the total and sampled points Zernike vectors for four binding regions. For each of the four considered proteins, the PCA of the Zernike vectors describing the interacting region is performed. The first column shows the projection on the first two PCs of the Zernike vectors of each point in the patch (in blue) and of the points selected with the optimal sampling (in red), with the respective marginal density distributions. The percentage of sampled over total points is reported in the legend. The bar plots in the second column show the percentage of points sampled with different sets of parameters; the bar in the box corresponds to the optimal set. In the third column the percentage of spanned PCs space as a function of the number of sampled points for different sets of parameters is shown. The last column reports the points in the PCs space colored according to the roughness of the centered patch.

The proposed sampling method is in principle applicable to any molecular surface, for example for the study of protein-protein interactions. In this case, to be of practical utility, the procedure should be able to characterize the binding regions of protein complexes efficiently and with a low computational cost, for evaluating the binding properties. To test whether our proposed algorithm gives a set of points which optimizes the information of the local geometry of the interacting regions, we look at the preservation of information in terms of Zernike vectors. To do so, we select the interacting regions of four protein surfaces, which are randomly selected from the dataset reported in [8], and define them as a set of vectors. Each vector corresponds to a point *i* of the interacting molecular surface and is composed of the Zernike coefficients obtained by centering the patch on that point. Next, we select only a subset of the points belonging to the interacting surfaces, in agreement with the proposed sampling algorithm, according to different parameters sets (having fixed *α* = 1 since in this case we have no interest in a uniform restriction of the number of selected points). We then perform a Principal Components Analysis (PCA) of the Zernike vectors associated to each binding site, in order to compact the information into the essential space composed of the first two principal components (whose explained variances are 0.54 0.57, 0.51 and 0.64). Fig 7 shows the surface points belonging to the binding site (in blue) and the subset selected by the algorithm with the optimal parameters (in red), for each one of the four surfaces. The fourth column shows the same points coloured according to their $R_{i}$; as expected, the sampling is more dense where the surface is more rough. Next, we compute the percentage of space occupied in the PCs plot by the selected points compared to that spanned by the total points. The results are shown in the third column of Fig 7. The optimal parameters samplings (marked in red) correspond to the situation from which a higher number of sampled points starts conveying less information in the representation of the binding regions in terms of the Zernike vectors space.

## Conclusion

In this paper, we present a new sampling method to reduce the number of points needed to describe a molecular surface. Given the importance of the definition of the molecular surface for the study of the interactions between molecules, we design a new algorithm to efficiently select the points of the original surface that minimize the loss of information in terms of describing the local shape of surface regions, which can potentially interact with a molecular partner. To test its performance, we use a recently developed method based on 2D *Zernike* polynomials, capable of describing the shape properties of portions of molecular surfaces. By means of *Zernike*, we verify if the patches centered around the sampled points are indeed the most representative of the surface. The sampling of these points is determined by Eq 7 and, when the best parameters are selected, results in a satisfactory reconstruction of the surface that needs only a subset of the total surface’s points.

To determine how accurate the method is in selecting a subset of surface points, we compare the results of the sampling with what is obtained with a random selection of the same number of points. This is done in terms of the mean distance between the *Zernike* vectors that describe a patch containing all the surface points and the ones that describe the same region, but containing this time only the points selected with the sampling or randomly.

We presented our novel sampling strategy on a case study of a small protein and then extended it on a set of 70 proteins, comparing the results coming from the optimal sampling with those obtained with the average value of the parameters.

Finally, to verify the applicability of our procedure, we showed the sampling ability to characterize binding sites of protein complexes in a set of cases. Thanks to the reduction of the points that have to be considered, the computational cost of the following analysis on the surfaces is reduced. Therefore, the method can be applied to (i) macromolecules composed of a high number of residues, (ii) the analysis of the molecular surface of frames obtained from molecular dynamics simulations, and (iii) molecular surfaces calculated at high resolution for which a high number of points is required for a more detailed description. Moreover, in principle the method can be adapted to other properties besides curvature, such as electrostatics or hydrophobicity, since these are described with numerical values, that can be assigned to each surface point. This would mean sampling the points maximizing the conservation of different properties (in addition to morphological ones) and we are working on this, since the Zernike formalism can be applied to describe any function.
